# Neuropeptide hormone bursicon mediates female reproduction in the whitefly, *Bemisia tabaci* (Hemiptera: Aleyrodidae)

**DOI:** 10.3389/fendo.2023.1277439

**Published:** 2023-10-03

**Authors:** Hao Yu, Bin Yang, Liuhao Wang, Sijia Wang, Kui Wang, Qisheng Song, Hongwei Zhang

**Affiliations:** ^1^ Department of Natural Resources, Henan Institute of Science and Technology, Xinxiang, Henan, China; ^2^ Division of Plant Science and Technology, University of Missouri, Columbia, MO, United States

**Keywords:** bursicon, reproduction, vitellogenesis, juvenile hormone, *Bemisia tabaci*

## Abstract

Bursicon, a neuropeptide hormone comprising two subunits-bursicon (burs) and partner of burs (pburs), belongs to the cystine-knot protein family. Bursicon heterodimers and homodimers bind to the lucine-rich G-protein coupled receptor (LGR) encoded by *ricket*s to regulate multiple physiological processes in arthropods. Notably, these processes encompass the regulation of female reproduction, a recent revelation in *Tribolium castaneum*. In this study we investigated the role of burs/pburs/rickets in mediating female vitellogenesis and reproduction in a hemipteran insect, the whitefly, *Bemisia tabaci*. Our investigation unveiled a synchronized expression of *burs, pburs* and *rickets*, with their transcripts persisting detectable in the days following eclosion. RNAi-mediated knockdown of *burs*, *pburs* or *rickets* significantly suppressed the transcript levels of *vitellogenin* (*Vg*) and *Vg receptor* in the female whiteflies. These effects also impaired ovarian maturation and female fecundity, as evidenced by a reduction in the number of eggs laid per female, a decrease in egg size and a decline in egg hatching rate. Furthermore, knockdown of *burs*, *pburs* or *rickets* led to diminished juvenile hormone (JH) titers and reduced transcript level of *Kruppel homolog-1*. However, this impact did not extend to genes in the insulin pathway or target of rapamycin pathway, deviating from the results observed in *T. castaneum*. Taken together, we conclude that burs/pburs/rickets regulates the vitellogenesis and reproduction in the whiteflies by coordinating with the JH signaling pathway.

## Introduction

Vitellogenesis stands as a critical phase within the female reproductive process in insects, holding significance in egg maturation and post-oviposition embryonic growth, thus determining the female fecundity in most cases (1). Vitellogenin (Vg) is synthesized in fat body (FB), released into hemolymph, and finally taken up by developing oocytes via receptor-mediated endocytosis. The receptor responsible for Vg uptake is a membrane-bound protein called Vg receptor (VgR), which is located on the surface of developmentally competent oocytes (2). In recent years, an increased focus has been placed on deciphering the molecular mechanisms regulating female vitellogenesis and reproduction. This stems from their importance in insect propagation and their potential as viable targets for pest control.

The current consensus strongly establishes that female reproduction including vitellogenesis, is regulated by the endocrine system. This intricate network encompasses hormones like juvenile hormone (JH), ecdysteroid hormone and various neuropeptides (3). JH is shown to be the predominant regulator controlling reproductive events, especially in hemimetabolous insect orders, such as Orthoptera (*Locusta migratoria*) (4), Blattodea (*Blattella germanica*) (5) and Hemiptera (*Nilaparvata lugens*) (6). In these insects, JH works through its receptor Methoprene-tolerant (Met) with another factor Taiman (Tai) to control vitellogenesis and oocyte maturation. This coalition triggers polyploidization of FB cells, induces *Vg* expression and facilitates ovarian development, among other influences (1, 3). In more advanced insects, JH’s role in regulating reproduction persists, yet it shares the spotlight with other vital hormones such as ecdysteroids, mainly 20-hydroxyecdysone (20E). This gives rise to a complex signaling network that depends on the specific reproductive traits of individual insect order or even species (7, 8). For instance, in the lepidopteran insect *Spodopetera frugiperda*, female reproduction is co-controlled by JH and ecdysteroids (9). In dipterans, JH mediates the developmental stage in preparation for the subsequent vitellogenesis, however, the leading role in regulating vitellogenesis and oogenesis is taken over by ecdysteroid (10, 11). Beyond the dual protagonists of JH and ecdysteroids, additional hormones and neuropeptides also contribute to the female reproductive process. It has been well established that the insulin-like peptide (ILP) and insulin pathways play a crucial role in regulating reproduction, given their vital functions in nutrient uptake essential for vitellogenesis, and in the biosynthesis of JH and ecdysteroids (12). Similar to the insulin pathway, the amino acid/target of rapamycin (AA/TOR) pathway also secures an essential role in ensuring reproductive success of insects (13). *Drosophila* insulin-like peptide 7 (DILP7) is essential for egg laying by controlling the ovipositor motor programs (14). Interestingly, hormones traditionally unlinked to reproduction also make their mark in this process. The ecdysis-triggering hormone (ETH), renowned for its role as a molting regulator, also functions as an essential allatotropin to promote JH synthesis and subsequently prime the reproduction process in *Drosophila* (15). Recent revelations from *Tribolium castaneum* shed light on the contribution of the neuropeptide bursicon in mediating reproductive physiology, particularly vitellogenesis. This function appears to be facilitated by the mediation of JH synthesis and interactions with the insulin/TOR pathways (16).

Bursicon, initially identified as a neuropeptide hormone responsible for insect cuticle tanning process post-molting, has now been revealed to have multifuctions in arthropod biology (17). Bursicon comprises two subunits: bursicon (burs or burs α) and partner of bursicon (pburs or burs β) (18). The interaction of bursicon heterodimer with its receptor, leucine-rich G protein-coupled receptor 2 (LGR2) in *Drosophila*, encoded by the gene *rickets*, stimulates the production of cAMP, which in turn, activates downstream cAMP-dependent kinase, to regulate specific biological processes (19). Bursicon has proven to be remarkably versatile, with diverse arrays of reported roles, encompassing functions such as initiating wing expansion (20), eliciting prophylactic immune responses (21, 22), regulating intestinal stem cell proliferation (23), and acting in migration of border cells during oogenesis (24). Among its diverse roles, bursicon’s novel function in mediating female vitellogenesis and reproduction was uncovered in *Tribolium castaneum* (16). This revelation led to the hypothesis that bursicon potentially exerts its influence on female reproduction in other insects. The mechanism underlying this process needs to be further elucidated. In this pursuit, we put our hypothesis to the test using the whitefly, *Bemisia tabaci*, one of the most destructive agricultural pests worldwide, as a model system. Herein, we present the findings arising from our investigation aimed at validating this hypothesis.

## Materials and methods

### Insects and sample preparation

The whiteflies (*B. tabaci*, MEAM1 strain) were reared on cotton plants in artificial climate chambers (Dongnan Instrument, Ningbo, China) and the environmental parameters were set as follows: temperature at 26°C, relative humidity at 60%, and photoperiod with 16 h: 8 h light:dark cycles. The purity of the population was confirmed by sequencing the mitochondrial *cytochrome oxidase I* (mt*COI*) gene to avoid contamination of different whitefly strains (25). For sample preparation, the whiteflies at different developmental stages, including egg, 1-4th instar nymph, and adult were collected and subjected to RNA extraction for subsequent analysis of target gene levels.

### Total RNA isolation and cDNA synthesis

For RNA isolation, the TRIzol reagent (Invitrogen, CA, USA) was used to extract the total RNA from each collected sample, and all protocols were carried out following the manufacturer’s instructions. Following DNase digestion to remove potential genomic DNA contamination, the quality of the isolated RNA was examined by agarose gel electrophoresis, while the RNA quantity was determined using a NanoDrop ND-1000 spectrophotometer (Thermo Fisher Scientific, MA, USA). Subsequently, the obtained RNA (2 μg) was utilized to synthesize the first-strand cDNA using the PrimeScript II 1^st^ Strand cDNA Synthesis Kit (Takara, Dalian, China), following the manufacturer’s protocols. The resulting cDNA samples were used as templates for following PCR reactions.

### Reverse transcription quantitative real-time PCR

Reverse transcription quantitative real-time PCR (qRT-PCR) was conducted to determine the expression levels of target genes using the QuantStudio 3 Real-time PCR system (Applied Biosystems, CA, USA). The qRT-PCR reaction mixture was prepared as follows: 10 μl of ChamQ SYBR Color qPCR Master Mix (Vazyme, Nanjing, China), 1 μl of cDNA template, 2 μl of primer pairs (2 μM for each forward and reverse primers) and nuclease-free water, making up a final volume of 20 μl. The primer sequences used in this study are listed in **Supplementary Table 1**, with the internal reference gene being the *tubulin* gene (26). The amplification program was set as follows: a single step of 95 °C for 3 min, followed by 40 cycles of 95 °C for 10 s and then 60 °C for 1 min. At the end of the cycles, the melting curve from 60 °C to 95 °C was recorded to confirm a single PCR product. The expression levels of target genes were calculated using 2^−ΔΔ^ *^C^*_T_ method (ΔΔ*C*_T  _ = Δ*C*_T-target gene_ − Δ*C*_T-tubulin_) (27).

### Bioinformatic analyses

The sequences of *burs*, *pburs* and *rickets* gene of *B. tabaci* were obtained from the gene database of the National Center for Biotechnology Information (NCBI). Protein prediction was executed through the ExPASy (http://www.expasy.ch/) translate tool. The conserved domains and motifs within these proteins were predicted by the Simple Modular Architecture Research Tool (SMART) (http://smart.embl-heidelberg.de/). The phylogenetic tree was constructed using the MEGA X software, with the neighbor-joining method at bootstrap of 1000 (28).

### Synthesis of double-stranded RNA

Sequence-specific primers with T7 promoter sequence (**Supplementary Table 1**) were used to amplify the selected sequences of *burs, pburs* and *rickets*. The resulting PCR products underwent confirmation and purification, subsequently serving as templates for the synthesis of dsRNA. The dsRNA was synthesized using the T7 High Yield RNA Transcription Kit (Vazyme, Nanjing, China), following the manufacturer’s instructions. Briefly, the synthesizing mixture consisted of 2 μl of 10 × transcription buffer, 2 μl of T7 RNA polymerase, 1 μg of the DNA template, 2 μl of each NTP, and RNase-free water, culminating in a total volume of 20 μl. The prepared mixture was then incubated at 37 °C for 2 h, followed by DNase I treatment to remove the template DNA. The synthesized dsRNA was further purified by phenol/chloroform extraction and ethanol precipitation, and dissolved in 30 μl of RNase-free water. The quality of generated dsRNA was examined by agarose gel electrophoresis and its quantity was determined on the NanoDrop ND-1000 spectrophotometer. In addition to the dsRNA targeting *burs, pburs* and *rickets*, a control dsRNA targeting a *GFP* gene was prepared using the same method mentioned above.

### RNA interference

A leaf-mediated RNAi method was employed to knockdown the levels of *burs, pburs* and *rickets* in the whiteflies, as described in a previous study (29). Briefly, a cotton leaflet was removed from the plant and placed in a 1.5-ml Eppendorf tube, containing 800 μl of dsRNA solution (0.5 μg/μl), while a piece of parafilm sealed the tube open end. This leaflet-containing Eppendorf tube was then inserted into a 50-ml tube, and the open end of the latter was sealed with cotton ball. The whiteflies (newly emerged female adults) were released into this system for RNAi, ingesting dsRNA through feeding on the leaflet which absorbs dsRNA from the solution. The dsRNA solution was replenished every two days. The efficiency of RNAi was confirmed by qRT-PCR at 24h post release.

At 72h post RNAi treatment, the expression levels of target genes in the whiteflies of different groups (dsburs, dspburs, dsrk and dsGFP) were determined by qRT-PCR. The selected target genes included: *Vg* and *VgR* for vitellogenesis; *Kruppel homolog-1* (*Kr-h1*) and *Met* in the JH signaling pathway; *insulin receptor* (*InR*) and *Akt serine/threonine kinase* (*Akt*) in the insulin/insulin-like signaling pathway; *target of rapamycin serine/threonine kinase* (*TOR*) and *ribosomal protein S6 kinase* (*S6K*) in the TOR pathway.

### JH titer analysis

At 72h post RNAi treatment, whiteflies from each group (dsburs, dspburs, dsrk and dsGFP) were collected and subjected to JH titer quantification, following a previously described method (30). For each sample, a total of 200 individuals were pooled and homogenized thoroughly using a plastic rod in a 1.5-ml Eppendorf tube with 100 μl of phosphate buffered saline (PBS, PH 7.4). The homogenized samples were centrifuged at 10,000 x *g* for 10 min at 4°C. The resulting supernatant (20 μl) was extracted to assess the JH titer, using an insect JH ELISA kit (Meilian Biotech, Shanghai, China), following the manufacturer’s instructions. Briefly, each sample was added into wells of a 96-well plate, and the specific HRP-conjugated antibody was then added and mixed gently. The plate was incubated at 37°C for 1 h and the mixture was discarded. The wells were washed with a washing buffer for 5 times, followed by adding with substrate A/B and incubated for 15 min. After incubation, a reaction stop buffer was added and the absorbance at 450 nm of each well was measured. The JH titer was calculated based on the standard curve generated using the provided standard samples within the kit. This experiment was performed with three technical repeats.

### Bioassay of female fecundity

The assessment of female fecundity after RNAi treatment (dsburs, dspburs, dsrk and dsGFP) involved the examination of ovarian development, as well as the quantification of egg number, size and hatch rate. To observe ovarian development, the ovaries were dissected from the female whiteflies and placed in pre-cooled PBS following a 5-day RNAi treatment. Imaged were captured using a stereomicroscope (Nikon SMZ745, Japan). For fecundity determination, 10 female whiteflies were used for each treatment group and the total number of eggs laid on the leaflet per female was counted over a span of 7 days. The experiment was repeated three times. The egg hatching rate was calculated as the percentage of eggs that successfully hatched compared to the total number of eggs laid. Egg size was measured and imaged using the stereomicroscope.

### Statistical analysis

To compare data among different experimental groups, One-way ANOVA followed by Tukey’s test was used to analyze the significant differences among these different treatment groups, and *p < 0.05* was accepted as statistically significant.

## Results

### Sequence analysis of *burs*, *pburs* and *rickets* genes from *B. tabaci*

Through a comprehensive exploration of the Whitefly Genome Database and the NCBI database, coupled with sequence BLAST and protein domain predictions, we identified specific transcripts from *B. tabaci*, including *burs* (NCBI reference number XM_019055262.1), *pburs* (NCBI reference number XM_019055040.1) and *rickets* (NCBI reference number XP_018911955.1). The nucleotide and amino acid sequences of *burs and pburs* genes were shown in **Supplementary Figure S1**. The burs protein consists of 154 amino acids, while the pburs protein comprises 175 amino acids. Both subunits belong to the cystine-knot protein family, with a similar structure to nerve growth factors (NGFs) in vertebrates. The *Bemisia rickets* gene encodes a protein recognized as a leucine-rich repeat-containing G protein-coupled receptor (LGR) for bursicon heterodimer and homodimer (**Supplementary Figure S2**). The burs, pburs and rickets are highly conserved across insect species examined, showcasing over 70% identity in their amino acid sequences. To elucidate their evolutionary context, we constructed phylogenetic trees encompassing burs, pburs and rickets proteins, along with selected sequences from other insect species. As shown in **Supplementary Figure S3**, most of these peptides were clustered in alignment with established insect taxa, suggesting an evolution conservation among burs, pburs and rickets genes.

### Expression profiles of *burs*, *pburs* and *rickets*

**Figure 1:**
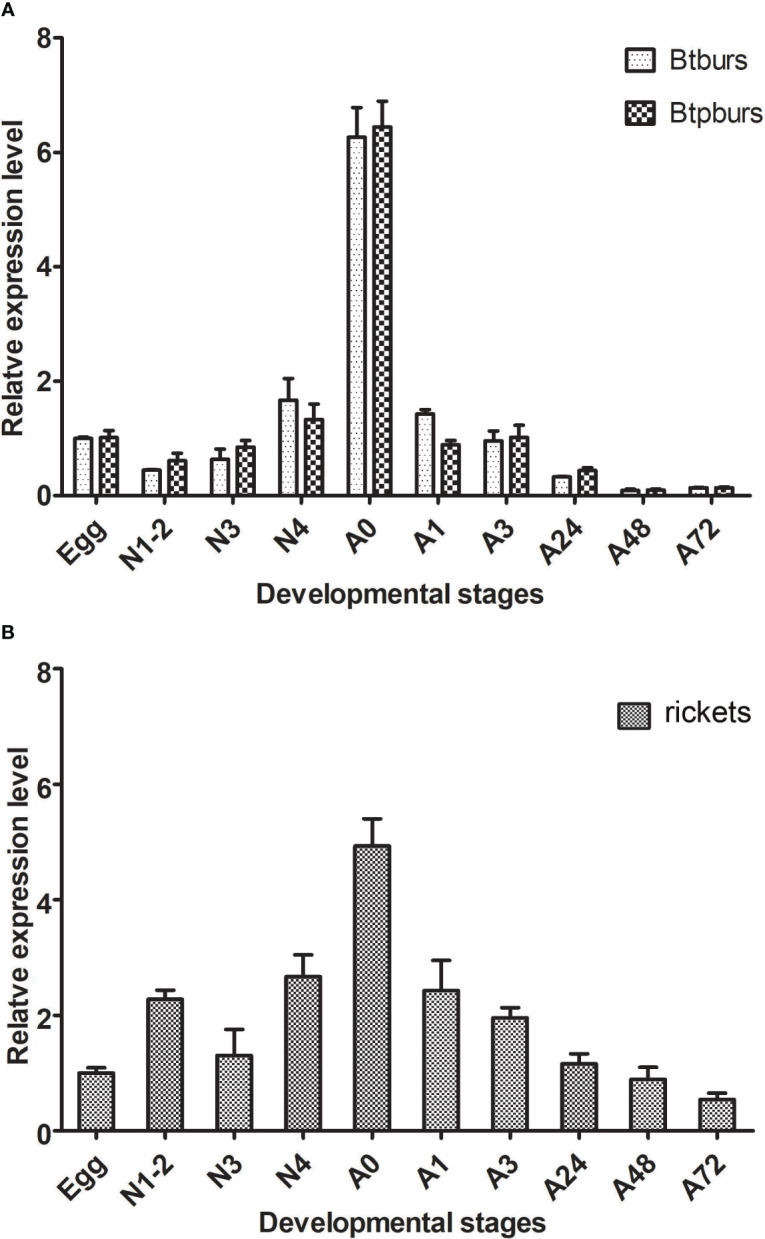
Temporal expression patterns of *burs and pburs* **(A)** and the receptor gene *rickets* **(B)** throughout the indicated life stages of the whiteflies. The histogram bars represent the average relative abundance of transcripts encoding each *burs, pburs* or *rickets*, with the error bars denoting a SD. This analysis was based on 3 independent biological replicates, and the data have been normalized to the gene expression level in eggs. The life stages are designated as follows: N1-2, 1st-2nd instar nymph; N3, 3rd nymph; N4, 4th instar nymph; A0, adult 0h; A1, adult 1h; A3, adult 3h; A24, adult 24 h; A48, adult 48h; A72, adult 72h.

The expression patterns of *burs*, *pburs* and *rickets* genes across different developmental stages of *B. tabaci* were determined by qRT-PCR. Results showed that *burs* transcripts remained constantly low throughout the developmental stages spanning from eggs to 4th instar larvae. However, a sharply increased *burs* level was observed in newly emerged adults. After eclosion, *burs* level gradually declined but remain detectable for at least 72 h (**Figure 1A**). The expression pattern of *pburs* was similar to that of *burs* (**Figure 1A**). Notably, transcripts of *rickets* gene were detected at all developmental stages, showing an ascending pattern from eggs to adults, followed by a subsequent decline post-eclosion (**Figure 1B**).

### Knockdown of *burs*, *pburs* or *rickets* suppressed the expression of *Vg* and *VgR*

**Figure 2:**
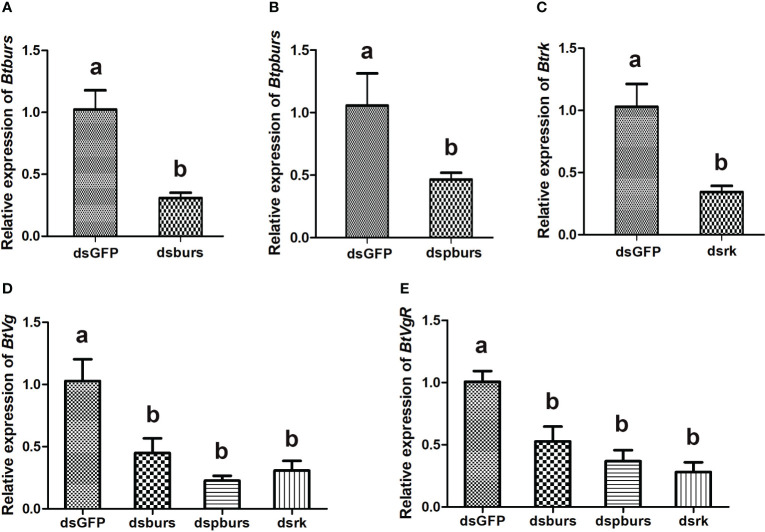
Expression profiles of indicated genes as detected by qRT-PCR. RNAi-mediated knockdown led to significant reduction in the relative expression levels of *burs* **(A)**, *pburs* **(B)** and *rickets* **(C)**. Knockdown of burs/pburs/rickets signaling genes affected the expression of *Vg* **(D)** and *VgR* **(E)** in female whiteflies. Data are represented as means ± SE and bars annotated with the same letter are not significantly different, *p* < 0.05.

To investigate the roles of burs/pburs/rickets signaling in regulating vitellogenesis, a leaf-mediated RNAi experiment was conducted. As shown in **Figure 2**, RNAi effectively suppressed the expression of *burs*, *pburs* or *rickets*, down by 50%-69% compared to the dsGFP control (**Figures 2A–C**). Knockdown of *burs*, *pburs* or *rickets* all led to decreased expression of *Vg* (**Figure 2D**). Particularly, *dspburs* exhibited the strongest effect, causing *Vg* expression down by 77%, compared with the dsGFP control (**Figure 2D**). Similarly, the expression of *VgR* was also suppressed upon the knockdown of these three genes, down by 47%-72% (**Figure 2E**).

### Knockdown of burs/pburs/rickets signaling genes impaired female fecundity

**Figure 3:**
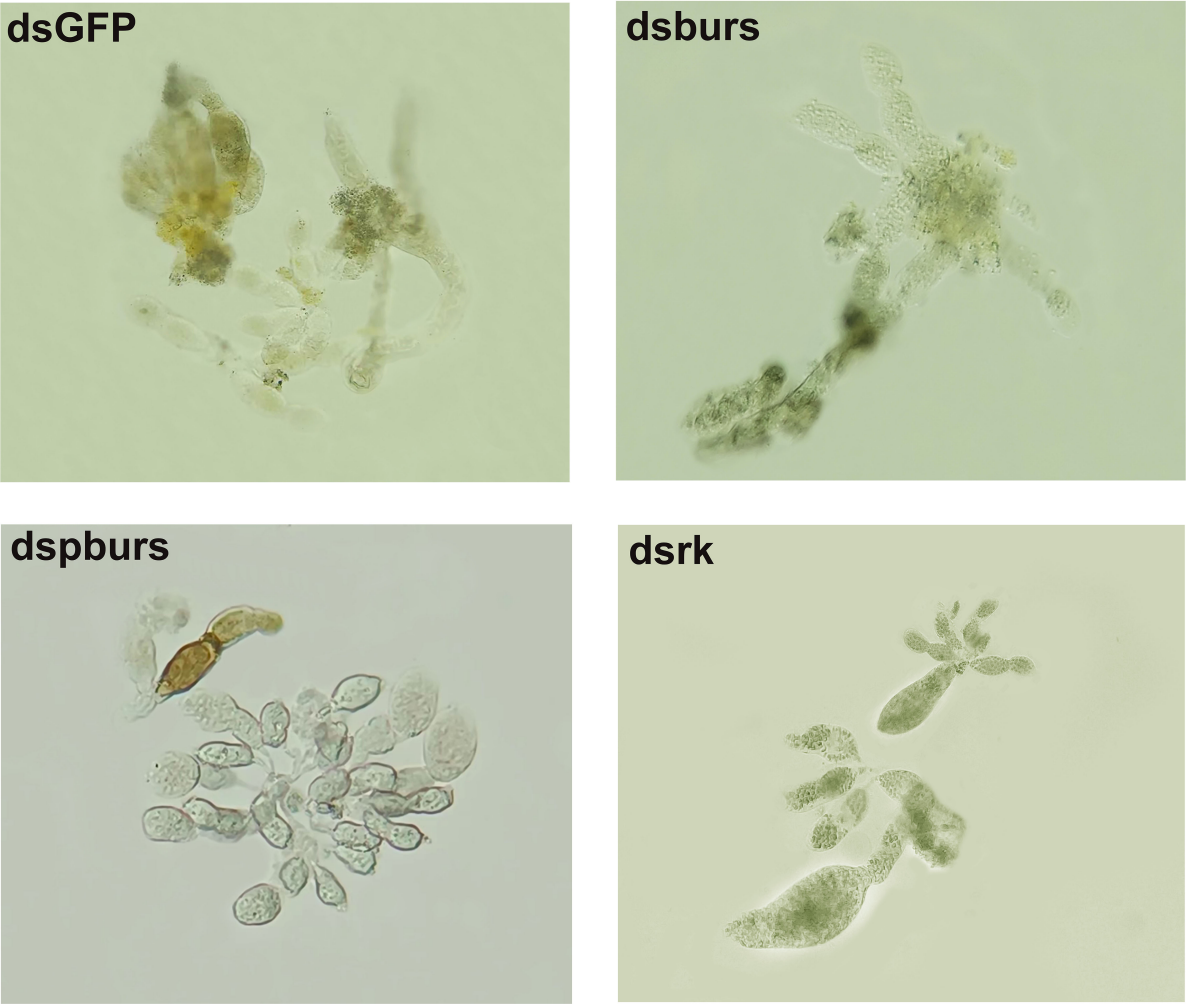
Ovarian phenotypes of female whiteflies after RNAi treatment with different dsRNAs, including dsburs, dspburs, dsrk and dsGFP.

Given the influence of burs/pburs/rickets signaling on the expression of *Vg* and *VgR*, we further examined whether the knockdown of *burs*, *pburs* or *rickets* could affect ovarian maturation in the female whiteflies. As shown in **Figure 3**, the dsGFP control group displayed a higher occurrence of fully developed oocytes, whereas the dsburs-, dspburs- or dsrk-treated groups exhibited a lower count of developed oocytes.

**Figure 4:**
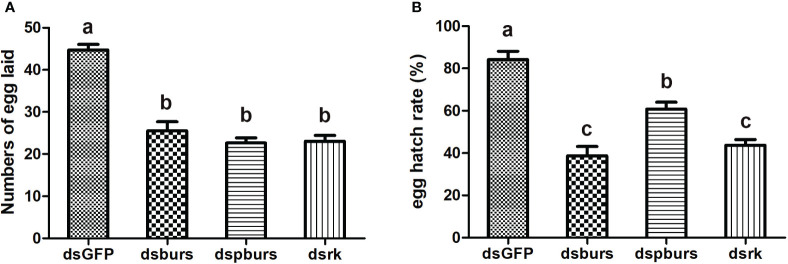
Effect of burs/pburs/rickets knockdown on female fecundity. **(A)** Knockdown of *burs*, *pburs* or *rickets* led to reduced numbers of egg laid per female. **(B)** Knockdown of *burs*, *pburs* or *rickets* decreased the egg hatch rates. Data are represented as means ± SE and bars annotated with the same letter are not significantly different. Significance is denoted by *p* < 0.05.

**Figure 5:**
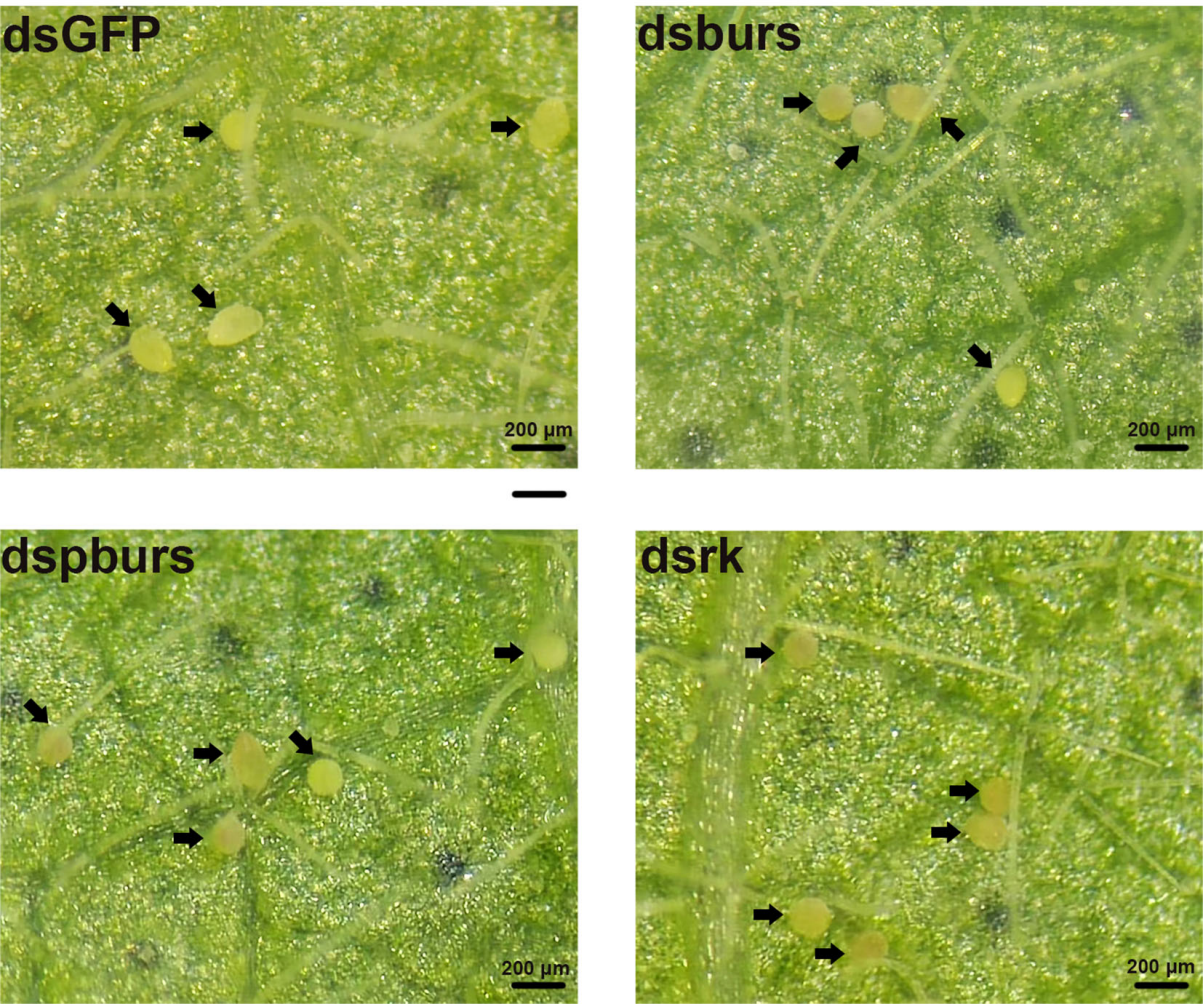
Phenotypes of the eggs laid by the female whiteflies after RNAi treatment of different groups (dsburs, dspburs, dsrk and dsGFP). Each egg on the leaf was indicated by a black arrow.

We proceeded to count the number of eggs laid by the female whiteflies treated with dsRNA and calculated the number of eggs per female laid in 7 days. Results showed that knockdown of burs/pburs/rickets signaling genes led to a notable reduction in egg production, with decreases by 43%, 49% and 48% for the dsburs, dspburs and dsrk groups, respectively (**Figure 4A**). The egg hatch rate was also calculated, showing a significant decrease by 54%, 28% and 48% respectively upon knockdown of *burs*, *pburs* or *rk* compared to the control group, with dspburs and dsrk displaying stronger effect compared to dsburs (**Figure 4B**). Moreover, we noticed that eggs laid by females in the dsburs, dspburs or dsrk groups were smaller in size compared to those in the dsGFP group (**Figure 5**).

### Burs signaling is correlated with JH signaling to regulate female reproduction

**Figure 6:**
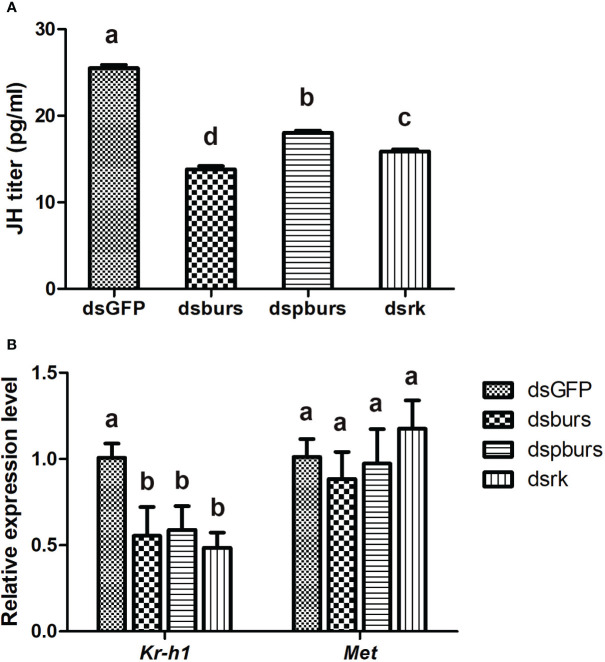
Effect of burs/pburs/rickets knockdown on JH titer and expression of JH signaling genes. **(A)** JH titers in the female whiteflies after knockdown of *burs*, *pburs* or *rickets*. **(B)** The expression levels of JH signaling genes, including the transcriptional factor *Kr-h1* and JH receptor *Met*, after knockdown of *burs*, *pburs* or *rickets*. Data are represented as means ± SE and bars annotated with the same letter are not significantly different. Significance is denoted by *p* < 0.05.

Since JH is the primary hormone governing female reproduction in Hemipterans, we investigated the potential interplay between burs/pburs/rickets signaling and JH signaling in regulating vitellogenesis. Firstly, we recorded the variations in JH titers after RNAi-mediated knockdown of *burs*, *pburs* or *rickets*. As shown in **Figure 6A**, the dsburs, dspburs and dsrk treated groups exhibited a significant reduction in JH titers compared to the dsGFP group, with the dsburs group showing the most significant decline. Secondly, we evaluated the impact of RNAi on the expression of genes in the JH signaling pathway. Results showed that the expression of *Kr-h1*, a JH-responsive gene, was downregulated upon knockdown of *burs*, *pburs* or *rickets*. However, the expression of *Met* remained unaffected by RNAi targeting these three genes (**Figure 6B**). Moreover, our findings indicated that knockdown of burs/pburs/rickets signaling genes did not affect the expression of genes associated with the insulin/TOR signaling pathways, including *InR*, *Akt*, *TOR* and *S6K* (**Supplementary Figure S4**).

## Discussion

The findings presented in this study provide robust evidence that bursicon plays a pivotal role in the female reproduction of *B. tabaci*, and potentially in other insect species as well. Several points lead to this conclusion. First, the sustained detectability of transcripts for burs, pburs and its receptor rickets post-eclosion strongly implies their involvement in adult development and reproduction. Second, knockdown of *burs*, *pburs* or *rickets* by RNAi significantly reduced the expression of *Vg* and *VgR* responsible for Vg uptake by developing oocytes. Third, knockdown of burs/pburs/rickets signaling genes impaired female fecundity, by affecting ovarian development, fecundity and egg hatch rate. Finally, burs/pburs/rickets signaling pathway was shown to mediate JH titers, the primary hormone regulating female reproduction in Hemipterans. Taken together, these findings substantiate our hypothesis that bursicon serves as a mediator of female reproduction in whiteflies, akin to its role reported in the case of another insect *T. castaneum*.

Neuropeptides and peptide hormones play a crucial role in regulating a wide range of developmental and physiological functions in insects, including reproductive processes (12). Previous studies have highlighted the significant influence of ILPs and insulin/TOR signaling in female insect reproduction, serving as nutrient sensors and coordinating with JH and ecdysteroids to form a complex regulatory network (3, 7). This has been demonstrated in various insect species, such as the American cockroach *Periplaneta americana* (31), the brown planthopper *Nilaparvata lugens* (6), the red flour beetle *T. castaneum* (32), and the yellow fever mosquito *Ae. aegypti* (13). Specifically, ILP and TOR signaling have been shown to be crucial for vitellogenesis and oocyte maturation. In *Drosophila*, DILP7 is responsible for initiating the ovipositor motor program, thereby controlling oviposition. Despite the well-studied ILPs, there are other reported cases that neuropeptides are capable of mediating vitellogenesis and reproduction (14). In *Drosophila*, ETH is involved in reproductive process by functioning as a link between 20E and JH signaling to regulate oogenesis and ovarian development (15). In *Ae. aegypti*, another neuropeptide, the ovary ecdysteroidogenic hormone plays a role in stimulating the ovaries to produce ecdysone (33). In *T. castaneum*, neuropeptide bursion has been implicated in coordinating reproduction through its interactions with JH and insulin/TOR signaling (16). Considering the complexity of hormone regulation network governing reproduction in insects, we speculate that additional roles of neuropeptides in this context await discovery.

Bursicon is a hormone composed of two subunits, burs and pburs, that can form either heterodimers or homodimers to function as effective hormones (18). In *Drosophila*, its receptor is a GPCR protein, a common feature for heterodimeric glycoprotein hormones. Our study investigated the expression of *burs*, *pburs* and their receptor *rickets* across different developmental stages of *B. tabaci*. We observed a similar expression pattern for *burs* and *pburs*, which is also found in other arthropod species. Although the expression level of *burs* and *pburs* have been reported to vary at certain points in other species (16, 21, 22), our findings show almost identical expression levels for these subunits. Moreover, our study revealed that *bursicon* transcripts remained detectable at least 72h post-eclosion in adult *B. tabaci*, contrasting with their rapid decline to undetectable levels in Dipterans including *D. melanogaster* and *Ae. aegypt*i (21, 22). Comparable findings were observed in *T. castaneum*, where bursicon transcripts were detectable at 24h post-eclosion (16). These differences in expression patterns among different insect species might indicate divergent roles for bursicon in adult physiological processes, including reproduction.

Our study confirmed the role of bursicon in regulating female reproduction and vitellogenesis in *B. tabaci*. RNAi-mediated knockdown of *burs*, *pburs* or *rickets* resulted in decreased expression of *Vg* and *VgR*, and adversely affected ovarian development and maturation. These findings corroborate earlier reports in *T. castaneum* (16) and the black tiger shrimp *Peneus mondon* (34). Moreover, we found that knockdown of burs/pburs/rickets signaling genes significantly impacted female fecundity in the whiteflies, evidenced by reductions in egg numbers, hatch rates and egg size. This aligns with expectations, as disruptions in Vg production, Vg uptake and ovarian maturation would logically affect the quantity and quality of eggs. Interestingly, in *T. castaneum*, the effect of *burs* RNAi on egg hatching rates was far more severe than that of *pburs* knockdown (16). However, in our study, the RNAi-mediated effects on egg numbers and hatching rates were comparable for *burs*, *pburs* and *rickets*. This suggests that the regulatory mechanisms of bursicon may vary among different species, influencing reproductive outcomes in distinct ways.

We further investigated the potential mechanisms through which bursicon regulates female fecundity in the whiteflies, focusing on its relationship with JH, the primary hormone governing female reproduction. Our results indicate that knockdown of burs/pburs/rickets signaling influenced JH signaling, resulted in reduced JH titer and downregulation of the downstream transcriptional factor *Kr-h1*. This suggests that bursicon works in coordination with JH signaling to regulate female reproduction. These findings are consistent with prior research in *T. castaneum*, where treatment with recombinant pburs protein significantly upregulated several genes in the JH signaling pathway (16). However, in contrast to its role in *Tribolium*, knockdown of *burs*, *pburs* or *rickets* in whiteflies did not affect the gene expression of insulin/TOR signaling pathways. The reason for this discrepancy is unclear, but it may reflect species-specific differences in reproductive signaling networks. We hypothesize that, in the whiteflies, bursicon might play a similar role to ETH in *Drosophila*, acting as a link between 20E and JH to establish a regulatory network (15), despite the structural differences between these two neuropeptides.

The heterodimeric structure of bursicon, composed of two subunits, is a characteristic feature of the cystine-knot protein family, which also includes transforming growth factors (TGFs) and vertebrate gonadotropins such as follicle-stimulating hormone (FSH) and luteinizing hormone (LH) (35). These hormones, characterized as heterodimeric glycoproteins, consist of two distinct subunits, each containing a conserved cystine-knot structure. In vertebrates, FSH and LH are well known for their roles in female reproduction by stimulating follicular development, production of steroid hormone and ovulation (36). However, no vertebrate gonadotropin-like glycoprotein has been identified in arthropods. The structural similarity between bursicon and other growth factors suggests its multi-faceted roles in growth, development, and reproductive processes. Multiple studies, including our own, support the role of bursicon in mediating female reproduction in arthropods by coordinating with the JH and, in some cases, insulin/TOR pathways (16, 34). It’s worth noting that our study used RNAi to knock down either burs or pburs, which means we cannot entirely rule out the contributions of bursicon homodimers and heterodimers to reproductive processes. Future investigations could benefit from exploring the roles of recombinant bursicon homodimers and heterodimers, or even overexpression experiments, to provide a more comprehensive understanding of bursicon function in reproduction.

## Data availability statement

The original contributions presented in the study are included in the article/**Supplementary Material**, further inquiries can be directed to the corresponding author.

## Ethics statement

The animal study was approved by research ethics committee of Henan Institute of Science and Technology. The study was conducted in accordance with the local legislation and institutional requirements.

## Author contributions

HZ: Conceptualization, Funding acquisition, Project administration, Writing – review & editing. HY: Investigation, Methodology, Writing – original draft. BY: Investigation, Writing – original draft. LW: Formal Analysis, Investigation, Writing – original draft. SW: Investigation, Writing – original draft. KW: Methodology, Writing – original draft. QS: Conceptualization, Writing – review & editing.
