# Supplementary material for: Neuropeptide hormone bursicon mediates female reproduction in the whitefly, Bemisia tabaci (Hemiptera: Aleyrodidae)
Source: Front Endocrinol (Lausanne). 2023 Oct 3;14:1277439. doi: 10.3389/fendo.2023.1277439 (PMC10579919; doi:10.3389/fendo.2023.1277439)
Supplement: Supplementary file 1 [file DataSheet_1.docx]

**Supplementary Information**

**Neuropeptide hormone bursicon mediates female reproduction in the whitefly, *Bemisia tabaci***

Hao Yu^1^, Bin Yang^1^, Liuhao Wang^1^, Sijia Wang^1^, Kui Wang^1^,

Qisheng Song^2^ and Hongwei Zhang^1,^ ^*^

^1^Department of Natural Resources, Henan Institute of Science and Technology, Xinxiang, Henan 453003, China

^2^ Division of Plant Science and Technology, University of Missouri, Columbia, Missouri 65201, USA

^⁎^ Corresponding author: Dr. Hongwei Zhang, Division of Natural Resources, Henan Institute of Science and Technology, Xinxiang, Henan 453003, China

Email: [zhwhtu@163.com](mailto:zhwhtu@163.com)

**Table S1.** Primers used in this study.

| Primers |  | Sequences 5’-3’ * |
| --- | --- | --- |
| *BursRTF* |  | AGATCCTACGCGCTTTCCAA |
| *BursRTR* |  | CCAGACCGTCCAAAACGAGA |
| *pBursRTF* |  | GGGAAGGATGTTGGCAGGAG |
| *pBursRTR* |  | GCCCACAGCACTATGGAAGA |
| *rkRTF* |  | AGAACAATTGAGGCCGTCGT |
| *rkRTR* |  | AAATGCTGCCCCTACCTTCC |
| *VgRTF* |  | ACGTTTGCACCTTTGCCTTC  GTGGAAAGCGCACTGTTGTT  GGCTGGCACTGTAGAAGTGT  GGCAATGACGAACATCAGGC |
| *VgRTR* |  | GTGGAAAGCGCACTGTTGTT |
| *VgRRTF* |  | GGCTGGCACTGTAGAAGTGT |
| *VgRRTR* |  | GGCAATGACGAACATCAGGC |
| *Kr-h1RTF* |  | AAGCCCTACTCCTGCGAGAT  GAGTTGAACGTCTCCGAGCA  GTGTCATCGGCAGTGGAGAA  CAGGAAAGTTGTGTCGGGGT |
| *Kr-h1RTR* |  | GAGTTGAACGTCTCCGAGCA  GTGTCATCGGCAGTGGAGAA  CAGGAAAGTTGTGTCGGGGT |
| *MetRTF* |  | GTGTCATCGGCAGTGGAGAA |
| *MetRTR* |  | CAGGAAAGTTGTGTCGGGGT |
| *TORRTF* |  | CGCGATCTTTGGCTGTGATG |
| *TORRTR* |  | TGCCATCAGGCTGTCTTTGT |
| *AktRTF* |  | AACAAGCGATTGGGAGGAGG |
| *AktRTR* |  | AACGCGTGTCTGTGTCTGAT |
| *InRRTF* |  | GTTCGCGATGGAGGGATCAT |
| *InRRTR* |  | TCATCATCGATGTCCGGCAG |
| *S6KRTF* |  | TTCACCTATGTTGCCCCGTC |
| *S6KRTR* |  | CGAGGTGAGCGAGCTTTGAT |
| *GFPiF* |  | TAATACGACTCACTATAGGGGCCAACCATTGTCACTACTT |
| *GFPiR* |  | TAATACGACTCACTATAGGGAGTATTTTGTTGATAATGGTCG |
| *BursiF* |  | TAATACGACTCACTATAGGGATGTTCAGGAGCTGGAA |
| *BursiR* |  | TAATACGACTCACTATAGGGCTATTCTGGCTTGGCAA |
| *PBursiF* |  | TAATACGACTCACTATAGGGCACCCCTTACGTCCTTT |
| *PBursiR* |  | TAATACGACTCACTATAGGGCTATCGAGAGTAATCGC |
| *rkiF* |  | TAATACGACTCACTATAGGGTAGCGAAGCTGGAACTTA |
| *rkiR* |  | TAATACGACTCACTATAGGGAGGAGCCTTTCCAGTCCTG |

*The T7 promotor sequence within RNAi primers are underlined.


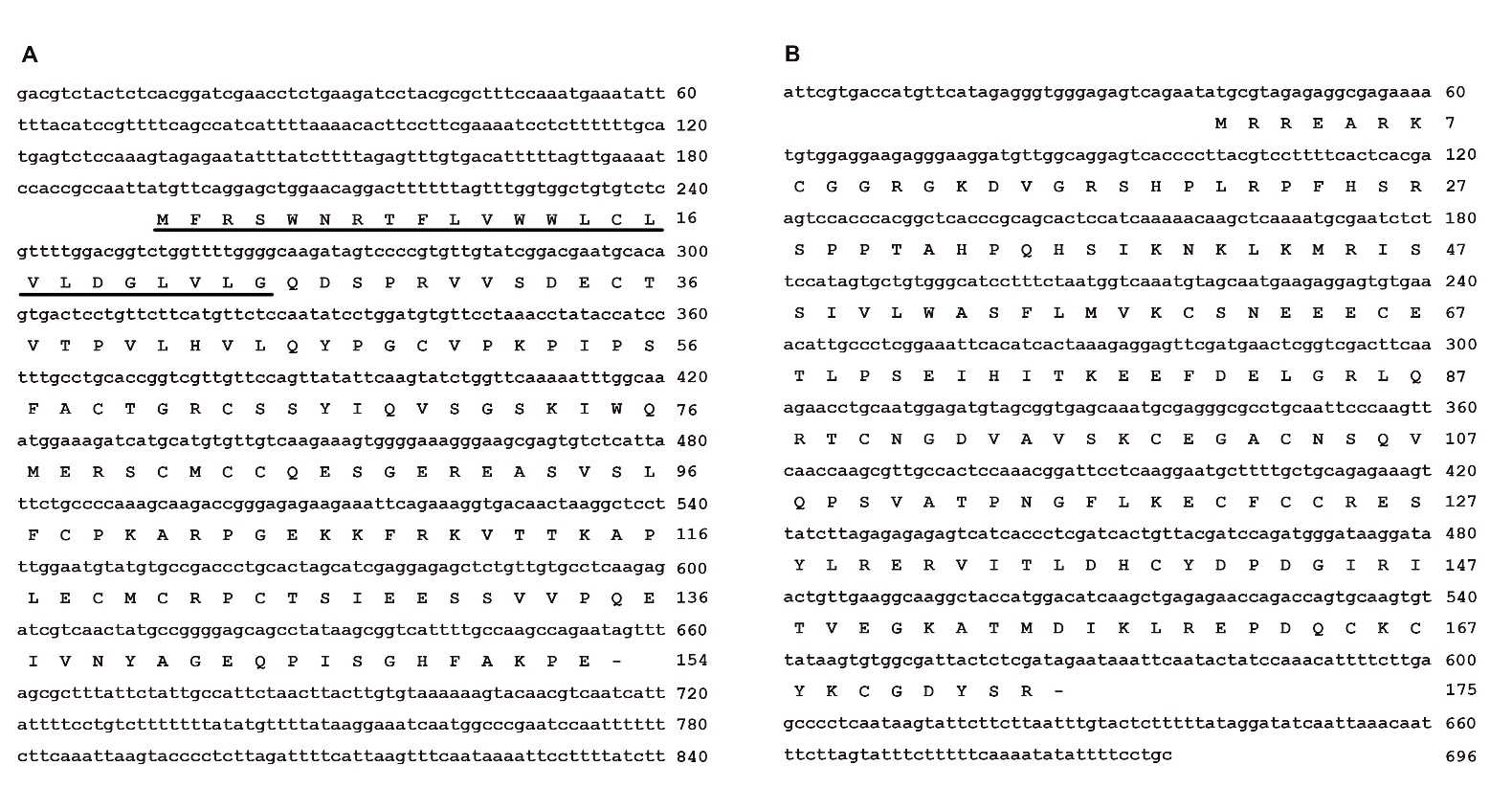


**Figure S1.** Nucleotides and deduced amino acid sequences of burs (A) and pburs (B) of *Bemisia tabaci*. The predicted signal peptides are underlined. Hyphens indicate stop codons.


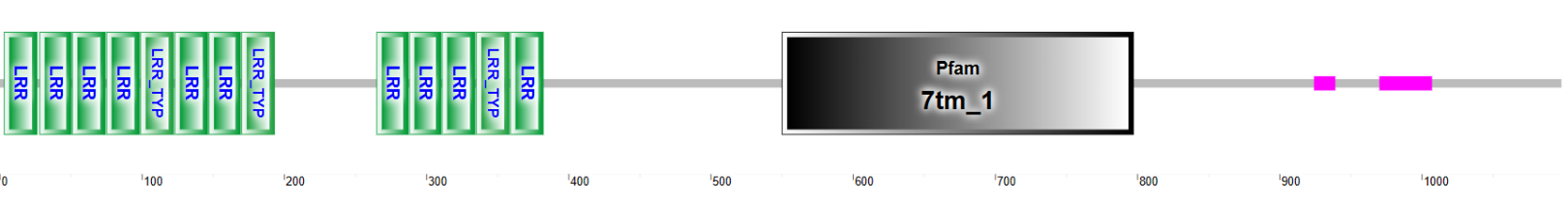


**Figure S2.** A schematic diagram of the structure of the bursicon receptor ricket protein, generated by Simple Modular Architecture Research Tool (SMART) (http://smart.embl-heidelberg.de/).

**
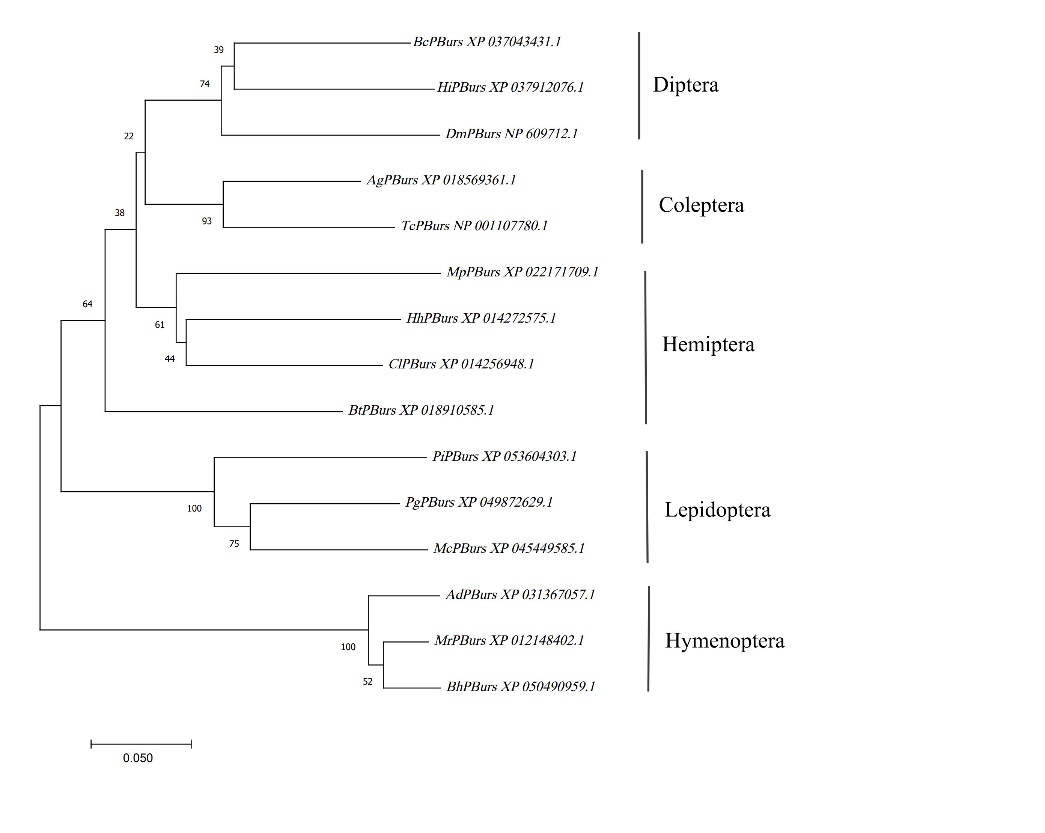
**
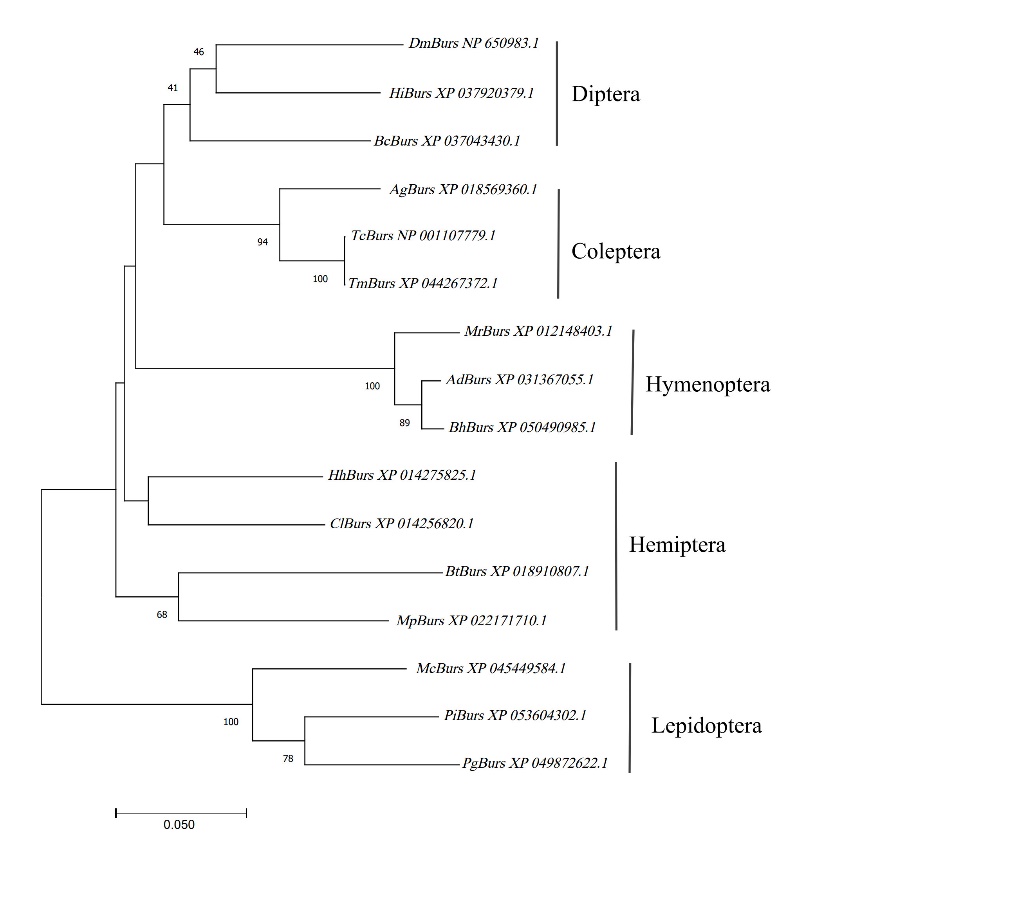
**A B**

**C**


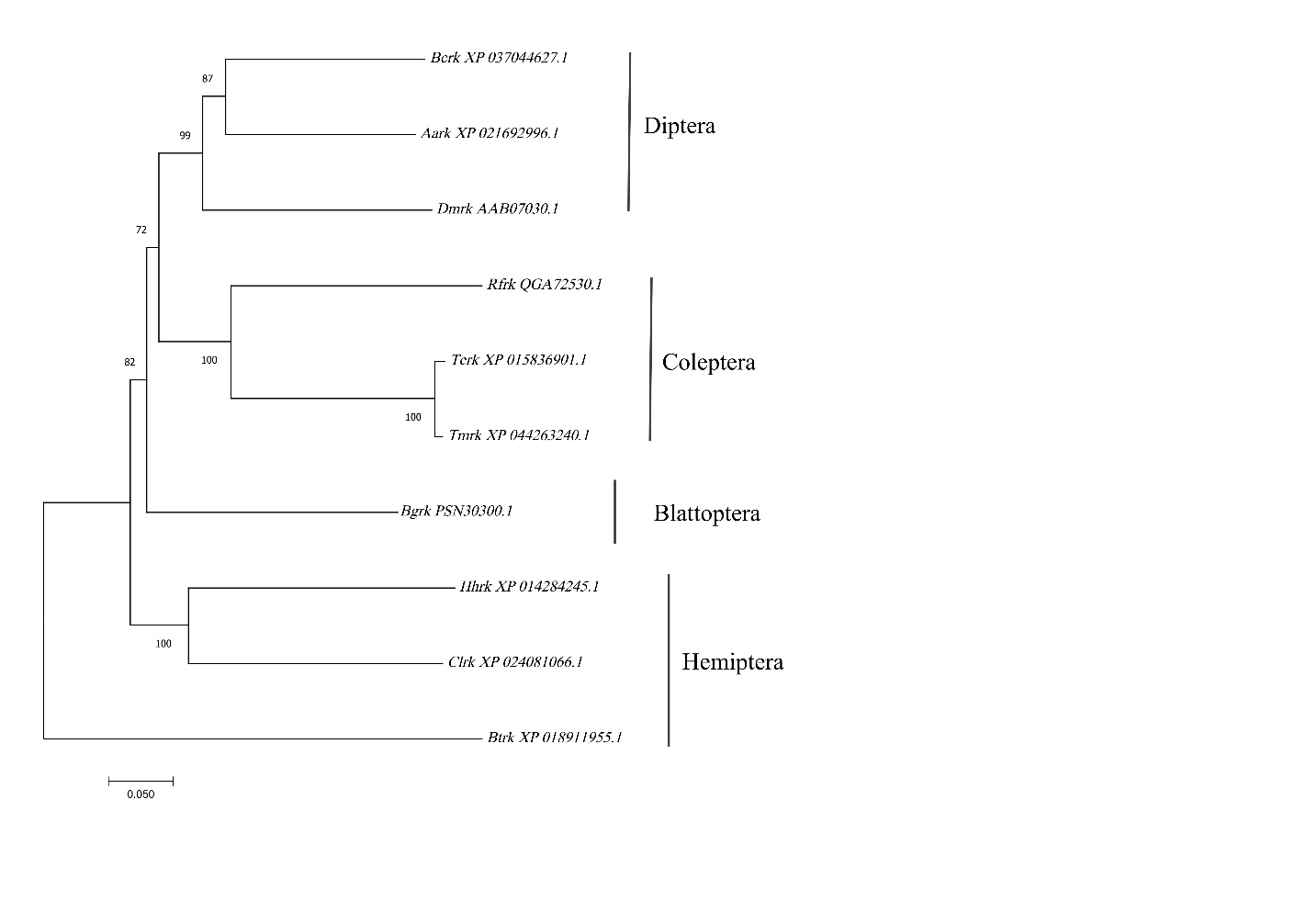


**Figure S3.** Phylogenetic tree based on the selected sequences of burs (A), pburs (B) and rickets (C) proteins from different insects. The bar (0.05) shows the genetic distance. Numbers at the nodes represent bootstrap proportions on 1000 replicates. The accession numbers of different proteins are labled on the tree.


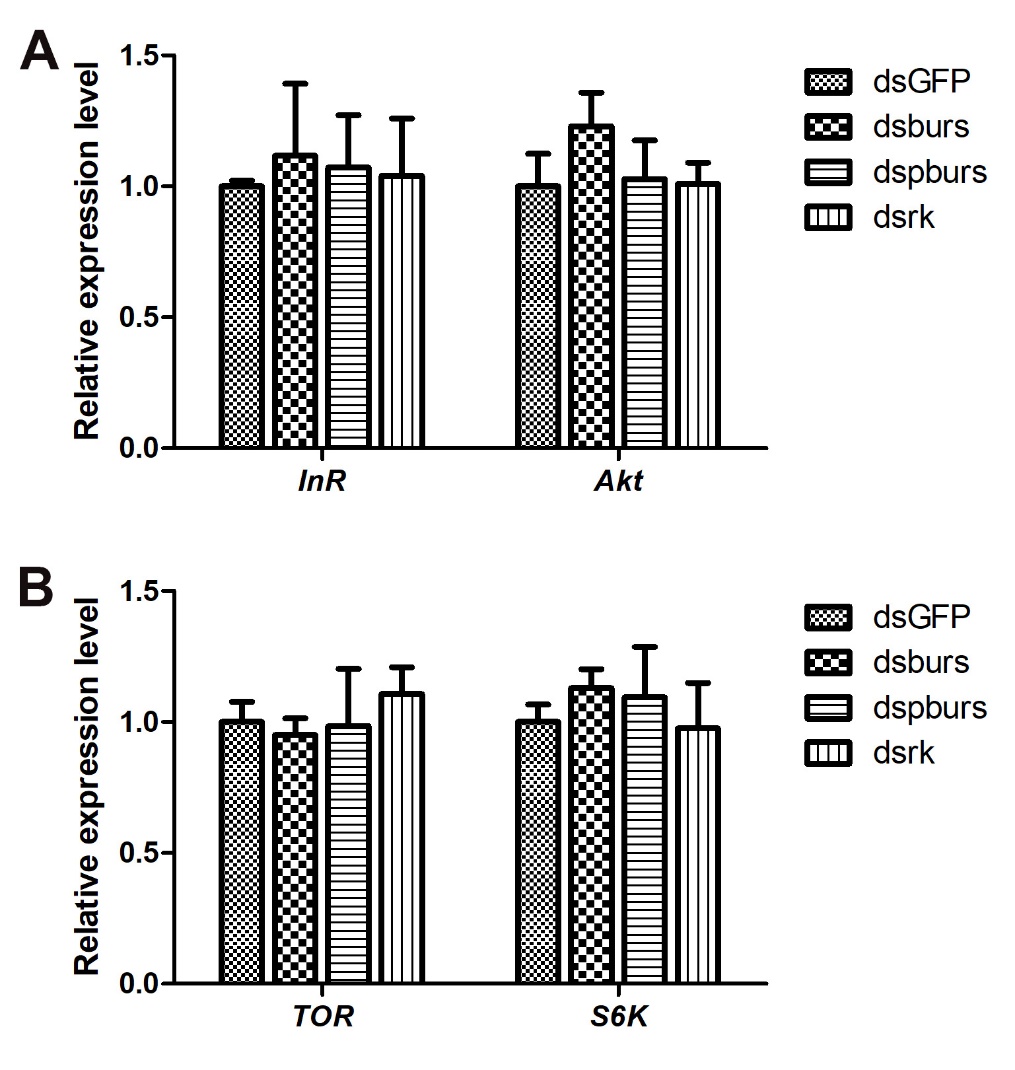


**Figure S4.** (A) Expression of insulin signaling genes, including the InR and Akt, after knockdown of *burs*, *pburs* or *rickets*. (B) Expression of TOR signaling genes, including *TOR* and *S6K*, after knockdown of *burs*, *pburs* or *rickets*. Data are represented as means ± SE and error bars indicate 1 SD, n = 3 independent biological replications.
